# Landscapes with different biodiversity influence distribution of small mammals and their ectoparasitic chigger mites: A comparative study from southwest China

**DOI:** 10.1371/journal.pone.0189987

**Published:** 2018-01-24

**Authors:** Pei-Ying Peng, Xian-Guo Guo, Dao-Chao Jin, Wen-Ge Dong, Ti-Jun Qian, Feng Qin, Zhi-Hua Yang, Rong Fan

**Affiliations:** 1 Institute of Entomology, Guizhou University, and the Provincial Key Laboratory for Agricultural Pest Management in Mountainous Region, Guiyang, P.R. China; 2 Vector Laboratory, Institute of Pathogens and Vectors, Yunnan Provincial Key Laboratory for Zoonosis Control and Prevention, Dali University, Dali, Yunnan Province, P.R. China; University of Minnesota, UNITED STATES

## Abstract

From a previous field investigation in Yunnan, southwest China between 2001 and 2015, we selected two types of landscapes to make a retrospectively comparative study on the distribution of small mammals and their ectoparasitic chigger mites. One landscape is “mountainous uncultivated land (MUL)” with higher biodiversity, which is located in a famous “World Nature Heritage Site”, the Three-Parallel-Rivers Region in the northwest of Yunnan. The other is “cultivated flatland landscape (CFL)” with lower biodiversity, which is located in the south of Yunnan. The landscapes with different biodiversity apparently influenced the distribution of small mammals and their ectoparasitic chigger mites. Much more species of small mammals and mites were found in MUL than in CFL. A total of 3,177 small mammals captured from MUL were identified as 55 species, 30 genera and 10 families in five orders. From these small mammal hosts, 5,882 chigger mites were collected and identified as 127 species, 15 genera and 3 subfamilies in two families. A total of 1,112 small mammals captured from CFL were identified as 19 species, 12 genera and 5 families in three orders. From these hosts, 17,742 chiggers were collected and identified as 86 species, 12 genera and 3 subfamilies in two families. Both the species diversity (*S* = 55) and community diversity (*H* = 2.673) of small mammals in MUL were much higher than those in CFL (*S* = 19; *H* = 0.926). There were also higher values of β diversity in MUL than in CFL. Different main reservoir rodent hosts of zoonoses (including tsutsugamushi disease) were found in two types of landscapes. *Rattus tanezumi* (one main reservoir host) was most abundant in CFL, which accounted for 80.22% of all the small mammals. Another two main reservoir hosts, *Eothenomys miletus* and *Apodemus chevrieri* were the dominant species in MUL, but they were not as abundant as *R*. *tanezumi* in CFL. Different vector species of chigger mites also existed in MUL and CFL. *Leptotrombidium deliense* (a main and powerful vector of tsutsugamushi disease in China) and *Ascoschoengastia indica* (a potential vector of tsutsugamushi disease) were the dominant species of chigger mites in CFL (*Cr* = 25.81% for *A*. *indica*; *C*_*r*_ = 23.47% for *L*. *deliense*). *Leptotrombidium scutellare* (also a main vector of tsutsugamushi disease in China) was the dominant chigger species in MUL (*Cr* = 26.09%). Higher infestation of vector mites on small mammals was found in the simple landscape with lower biodiversity (CFL) than in the complex landscape with higher biodiversity (MUL). The overall prevalence (*P*), mean abundance (*MA*) and mean intensity (*MI*) of chigger mites on small mammals were much higher in CFL than in MUL. The main vector mite species on their main rodent hosts also showed a higher *P*, *MA* and *MI* in CFL than in MUL.

## Introduction

Small mammals usually involve five orders, Rodentia, Insectivora, Scandentia, Lagomorpha and Chiroptera. Of the five orders, the majority of small mammals are rodents (rats, mice, voles and squirrels, etc.) with more than 2000 species recorded globally [1]. Small mammals are an important constituent part in natural ecosystems and food webs (food chains). They are of great significance in maintaining the integrity of ecosystem and ecological equilibrium [1, 2]. Besides destroying crops in agriculture, some rodents can be reservoir hosts for zoonotic diseases and play a crucial role in maintaining and transmitting diseases [3, 4]. The Asian house rat, *Rattus tanezumi* (also called *Rattus flavipectus*), for example, is the main animal host and infection source of scrub typhus, plague and some other zoonoses in the foci [5, 6].

Chigger mites (trombiculid mites) are a large group of arthropods, which belong to two families (Trombiculidae and Leeuwenhoekiidae) in Acari [7]. There are several stages in the life cycle of chigger mites, but only the larva lives as ectoparasitic stage on a host’s body. Rodents and other small mammals are the most common hosts of the larvae of chigger mites [7, 8]. The larvae of chigger mites can transmit the pathogen of scrub typhus, *Orientia tsutsugamushi*, through their biting activity [7, 9, 10]. Besides transmitting scrub typhus, some chigger mites have been proven to be the potential vectors of hemorrhagic fever with renal syndrome caused by hanta virus [11–13].

Because of the medical importance of small mammals and their ectoparasitic chigger mites, it is worth understanding their distribution in different geographical regions and landscapes with different biodiversity. There have been some studies reporting the relationship between biodiversity and human health. Some reports suggested that simplified landscapes and biodiversity loss could increase the incidence of some infectious diseases [14–24]. Some experts pointed out that urban green space could increase the biodiversity of a city and its surrounding areas, and this could combat many urban ills [25–27]. Based on previous studies, we hypothesize that landscapes with different degrees of biodiversity would influence the distribution of small mammals and their ectoparasitic chigger mites. In comparison with complex landscapes, the species diversity of small mammals and chigger mites would be lower in the simple landscapes, but the individual abundance of some reservoir rodent species and vector chigger mite species would be higher in simple landscapes. To test the hypotheses, we selected two types of landscapes in Yunnan province, southwest China from our previous field investigation and made a retrospectively comparative study on the distribution of small mammals and their ectoparasitic chigger mites. One type of landscape is the mountainous uncultivated land (MUL) with higher biodiversity, a complex landscape, which is located in a famous “World Nature Heritage Site”, the Three-Parallel-Rivers Region in the northwest of Yunnan. The Three-Parallel-Rivers Region where MUL was selected is also known as a “biological gene bank of the world” [28]. The other type of landscape is the cultivated flatland (CFL) with lower biodiversity, a simple landscape, which is in the south of Yunnan. The south of Yunnan where CFL was selected have a wide scope of flatland-type farmlands, which is far from the northwest of Yunnan where MUL was selected. Yunnan province in southwest China, where the above MUL and CFL were selected, is an ideal place to study biodiversity, because the acreage of the province accounts for less than 0.4% of the whole China, but it has more than 20% of higher plants and 25% of animal species of the whole country [29–31].

In the present study, the original data in both MUL and CFL came from a part of the previously accumulated field investigation between 2001 and 2015 [32–34]. In each landscape of MUL and CFL, six counties were selected for the study. The analysis of the present paper by using a part of the previous investigation data belongs to the “data mining” process, which aims to elucidate some new phenomena and distribution patterns within some existed data.

## Materials and methods

### Study sites

As mentioned above, two types of landscapes (MUL and CFL) in Yunnan province of southwest China were selected from our previous field investigation between 2001 and 2015[32–34]. In each landscape of MUL and CFL, six counties were selected for the study. The distribution of small mammals and their ectoparasitic chigger mites was retrospectively studied between these two landscapes, MUL and CFL.

The mountainous uncultivated land (MUL) with higher biodiversity, a complex landscape in the Three-Parallel-Rivers Region of northwest Yunnan, involved a complex type of wild habitats with various kinds of plants and higher biodiversity, including bush fallows, second growth woodlands, wild grasslands and some other wastelands. From low latitude to high latitude, the following six counties (each county with only one selected investigation site) in MUL were selected for the study: Fugong, Lijiang, Weixi, Gongshan, Xianggelila and Deqin.

**Fig 1:**
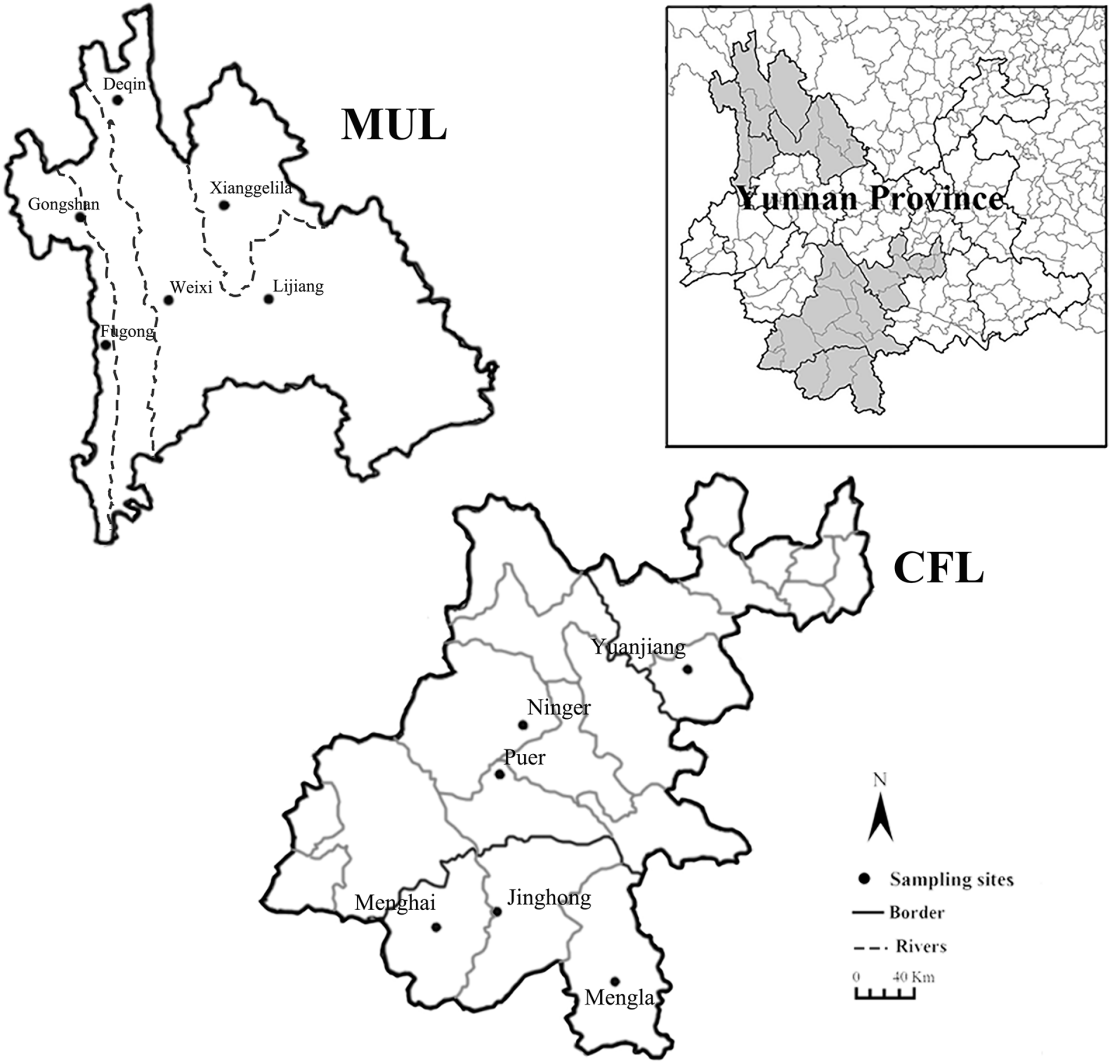
The study sites in the mountainous uncultivated areas (Three Parallel Rivers Region) (MUL) and the cultivated flatlands (CFL) from 2001 to 2015.

The cultivated flatland (CFL) with lower biodiversity, a simple landscape in the south of Yunnan mainly involved a simple type of habitats, farmlands (paddy fields and non-irrigated farmlands) with lower biodiversity. From low latitude to high latitude, the following six counties in CFL were selected for the study: Mengla, Menghai, Jinghong, Puer, Ninger and Yuanjiang. There was only one selected investigation site in each of five counties (Mengla, Menghai, Puer, Ninger and Yuanjiang) in CFL, but two investigation sites in Jinghong (Jinghong site and Jingha site) which is an exception (Fig 1).

### Collection and identification of chigger mites and their hosts

In our previous field investigation between 2001 to 2015, small mammals (mainly rodents) were sampled from each study site in two types of landscapes, MUL and CFL (Fig 1). The small mammals were mainly trapped with mousetraps (Guixi Mousetrap Apparatus Factory, Guixi, Jiangxi, China) from different habitats of each study site. Mousetraps (18cm×12cm×9cm) baited with corns, peanuts or some other food baits, were set in different types of habitats (bush fallows, woodlands, grasslands, paddy fields and some other farmlands) in the evening and checked the following morning. A total of 3000 traps were accumulatively placed in various types of habitats in each investigation site (about 200 traps/per night), which usually lasted about 15 days for each site of investigation.

The trapped rodents and other small mammals (hosts) were separately placed in pre-marked white cloth bags and then transferred to the laboratory for the collection of chigger mites. In the laboratory, each trapped host was anaesthetized with ether (cotton balls soaked with ether) within a close container. Most harmful rodents to agriculture (pests) were euthanized to death in deep anesthesia (euthanasia). (ether was used as a form of euthanasia in our manuscript). Some non-pest small mammals (weasels and moles, for example), however, were anaesthetized only for two to five minutes according to their body size, and these hosts were marked with yellow dye under the anesthesia (fur staining method)[35]. Most rodents were considered "pests" in agriculture and the local government encourage people to kill and eradicate them. All the "pests" rodents were euthanized to death according to the above methods. (Please see the Appendix at the end of the paper about the full list of species that were considered "pests" and the number that were euthanized). The systematic collection of chigger mites was done under the anesthesia of the hosts. The marked hosts were released to the wild after they waked up. Over a large white tray and with the help of a magnifier, the larvae of chigger mites were mainly collected by a curette (or earpick) and lancet from the auricles and external auditory canals of the host ears, which are the most common parasitic places of chigger larvae [4, 7]. In addition, the groin, perineal and axillary regions of the hosts were also examined as well, where the larvae are probably attached. Few recaptured hosts previously marked were not repeatedly examined. The collected chigger mites from each host were separately preserved in labeled vials containing 70% ethanol [4, 7, 8]. After collection, the white plate and other instruments were cleaned with disposable paper towels to reduce the chance of cross-contamination. The collected chiggers were made into glass slide specimens before identification. Chigger mites were rinsed with clean water to remove the ethanol and mounted on glass slides with Hoyer’s medium. All the mounted chigger specimens were finally identified to species under a microscope [4, 7]. The capturing of small mammals was officially permitted by the local authority of wildlife service in Yunnan Province, China. The use of animals (including rodent euthanasia) for research was also officially approved by Ethics Committee of Dali University. The specimens of voucher chigger mites and representative small mammals are deposited in the specimen repository of the Institute of Pathogens and Vectors, Dali University, China.

### Statistical analysis

For each species of small mammal hosts and their ectoparasitic chigger mites, the constituent ratio (*Cr*), prevalence (*P*), mean abundance (*MA*) and mean intensity (*MI*) were calculated [36]. Species richness (*S*) and Shannon-Wiener diversity index (*H′*) were adopted to analyze community structure of small mammals and chigger mites [37–39]. The Cody diversity index was used to measure the Beta-diversity (β diversity) estimation of hosts and chiggers [40]. The calculation formulae are as follows.

$$\begin{array}{llll} C_{r}=\left(\frac{N_{i}}{N}\right)\times 100\%; & P=\left(\frac{H_{i}}{H}\right)\times 100\%; & MA=\frac{N_{i}}{H}; & MI=\frac{N_{i}}{H_{i}}; \end{array}$$

$$\begin{array}{lll} S=\sum S_{i}; & H^{\prime }=-\sum_{i=1}^{S}\left(\frac{N_{i}}{N}\right)\text{ln}\left(\frac{N_{i}}{N}\right); & \beta_{C}=\left[g\left(h\right)+l\left(h\right)\right]/2 \end{array}$$

In the above formulae, *N*_*i*_ represents the individuals of host species (small mammal) *i* or chigger mite species *i*. *N* represents the total individuals of all species of small mammals or chigger mites. *H* represents the total number of individual small mammal hosts. *H*_*i*_ represents the number of individual hosts infested by chigger mite *i*. *S*_*i*_ represents the number of small mammal species *i* or chigger mite species *i*. The *g*(*h*) is the number of species that increase along the habitat gradient *h*. The *l*(*h*) is the number of species that decrease along the habitat gradient *h*. The Chi-square test was adopted to analyze the percentages of rodent species and individuals in two types of landscapes.

## Results

### Species composition and diversity of small mammals in two types of landscapes

The results showed that the species composition and diversity of small mammals were very different in two types of landscapes. In MUL with higher biodiversity in the Three Parallel Rivers region of Yunnan, a total of 3,177 small mammals were captured between 2001 and 2015, and they were identified as 55 species, 30 genera and 10 families in five orders: Rodentia, Insectivora, Scandentia, Lagomorpha and Carnivora (Table 1). In CFL with lower biodiversity in the south of Yunnan, a total of 1,112 small mammals were captured in the same period of time (2001–2015) and they were identified as 19 species, 12 genera and 5 families in three orders: Rodentia, Insectivora and Scandentia (Tables 2 and 3). As showed in Table 3, both the species diversity (*S* = 55) and community diversity (*H* = 2.673) of small mammals in MUL were much higher than in CFL (*S* = 19; *H* = 0.926).

**Table 1:** Small mammal hosts captured in MUL from 2001 to 2015.

Taxonomic taxa of small mammal hosts	Captured species and individuals of small mammal hosts (The figures in the brackets are the collected individuals for each host species)
**Rodentia**	Total hosts: 2,766 individuals; 39 species, 18 genera, 4 families
Muridae	*Apodemus draco* (641); *A*. *chevrieri* (410); *A*. *deninsulae* (104); *A*. *sylvaticus* (15); *A*. *latronum* (233); *A*. *agrarius* (4); *Rattus tanezumi* (89); *R*. *nitidus* (98); *R*. *norvegicus* (69); *R*. *sladeni* (28); *R*. *bowersi* (12); *Niviventer confucianus* (231); *N*. *fulvescens* (101); *N*. *andersoni* (10); *N*.*eha* (1); *N*. *excelsior* (2); *Mus caroli* (1); *M*. *pahari* (4); *M*. *musculus* (4); *Micromys minutus* (1); *Vernaya fulva* (1); *Vandeleuria oleracea* (1); *Bandicota indica* (1); *Leopoldamys edwardsi* (3).
Cricetidae	*Eothenomys miletus* (506); *E*. *eleusis* (1); *E*. *proditor* (60); *E*. *custos* (56).
Sciuridae	*Callosciurus erythraeus* (2); *C*. *quinquestriatus* (4); *Tamiops swinhoei* (7); *Dremomys pernyi* (20).
Petauristidae	*Petaurista clarkei* (6); *P*. *albiventer* (4); *P*. *xanthotis* (7); *Trogopterus xanthipes* (12); *Pteromys volans* (11); *Hylopetes alboniger* (4); *Belomys pearsonii* (2).
**Insectivora**	Total hosts: 369 individuals; 12 species, 9 genera, 3 families
Erinaceidae	*Neotetracus sinensis* (1).
Talpidae	*Scaptonyx fusicaudus* (6); *Nasillus gracilis* (5).
Soricidae	*Suncus murinus* (17); *Crocidura attenuata* (14); *Anourosorex squamipes* (271); *Soriculus leucops* (21); *S*. *caudatus* (2); *Sorex cylindricauda* (4); *S*. *bedfordiae* (13); *S*. *excelsus* (13); *Crocidura dracula* (2).
**Scandentia**	Total hosts: 1 individuals; 1 species, 1 genera, 1 family
Tupaiidae	*Tupaia belangeri* (1).
**Lagomorpha**	Total hosts: 40 individuals; 2 species, 1 genera, 1 family
Ochotona	*Ochotona thibetana* (36); *O*. *roylei* (4).
**Carnivora**	Total hosts: 1 individual; 1 species, 1 genera, 1 family
Mustelidea	*Mustela kathiah* (1).

**Table 2:** Small mammal hosts captured in CFL from 2001 to 2015.

Taxonomic taxa of small mammal hosts	Captured species and individuals of small mammal hosts (The figures in the brackets are the collected individuals for each host species)
**Rodentia**	Total hosts: 1,069 individuals; 14 species, 8 genera, 2 families
Muridae	*Rattus tanezumi* (892); *R*. *nitidus* (6); *R*. *norvegicus* (19); *R*. *sladeni* (68); *R*. *bowersi* (4); *Nivivente*r *confucianus* (14); *N*. *fulvescens* (48); *Mus pahari* (7); *M*. *musculus* (4); *Apodemus chevrieri* (1); *Micromys minutus* (2); *Leopoldamys edwardsi* (1); *Berlmys berdmoei* (2).
Cricetidae	*Eothenomys eleusis* (1).
**Insectivora**	Total hosts: 35 individuals; 4 species, 3 genera, 2 families
Erinaceidae	*Neotetracus sinensis* (2);
Soricidae	*Suncus murinus* (21); *Crocidura attenuata* (10); *C*. *dracula* (2).
**Scandentia**	Total hosts: 8 individuals; 1 species, 1 genera, 1 family
Tupaiidae	*Tupaia belangeri* (8).

**Table 3:** The comparative analysis of small mammal hosts captured from MUL and CFL (2001–2015).

		MUL			CFL
Orders		5			3
Families		10			5
Genera		30			12
Species (*S*)		55			19
Individuals		3,177			1,112
Shannon–Wiener diversity index (*H*)		2.673			0.926
Dominant small mammal hosts	Names	*A*. *draco*	*E*. *miletus*	*A*. *chevrieri*	*R*. *tanezumi*
	Individuals	641	506	410	892
	*C*_*r*_ (%)	20.176	15.927	12.905	80.216

### Distribution difference of reservior rodents in two types of landscapes

Of the captured small mammals (55 species and 3,177 individuals) in MUL, rodents accounted for 70.91% of species (39/55) and 87.06% of individuals (2766/3177) (Table 1). Of 19 species and 1,112 individuals of small mammals in CFL, the constituent ratios of rodent species and individuals reached 73.68% (14/19) and 96.13% (1069/1112) (Table 2). The percentages of rodent species and individuals in CFL were higher than those in MUL (P<0.05, P<0.01).

Different main rodent species of reservoir hosts of zoonoses were found in two types of landscapes. As one main reservior host of tsutsugamushi disease and some other zoonoses, the Asian house rat (*R*. *tanezumi*) was the most abundant species of small mammals in CFL, which accounted for 80.22% of all the captured small mammals. Another two main reservoir hosts, the Yunnan red-backed vole (*E*. *miletus*) and Chevrier’s field mouse (*A*. *chevrieri*) were the dominant species in MUL, which accounted for 15.93% and 12.91% of all the captured small mammals, but they were not as abundant as *R*. *tanezumi* in CFL.

### Species composition and diversity of chigger mites in two types of landscapes

In MUL with higher biodiversity in the Three Parallel Rivers region of Yunnan, a total of 5,882 chigger mites were collected from 3,177 small mammal hosts, and they were identified as 127 species (not including 90 chigger mites unidentified), 15 genera, 3 subfamilies (Trombiculinae, Gahrliepiinae, Leeuwenhoekiinae) in two families (Trombiculidae and Leeuwenhoekiidae). The subfamily Trombiculinae had the most abundant species and individuals (96 species in 8 genera with 4,482 individuals), and Gahrliepiinae came next (27 species in 4 genera with 1,283 individuals). Leeuwenhoekiinae had the least species and individuals (4 species in 3 genera with 27 individuals) (Table 4). As shown in Table 5, three species of chigger mites (*L*. *scutellare*, *G*. *fimbriata* and *L*. *densipunctatum*) were the most dominant in MUL and their constituent ratios (*C*_*r*_) were 26.10%, 13.47% and 9.01% respectively.

**Table 4:** Chigger mites collected and identified in MUL from 2001 to 2015.

Taxonomic taxa of chigger mites	Collected individuals and species of chigger mites (The figures in the brackets are the collected individuals for each mite species)
Trombiculidae	5,765 individuals; 123 species, 12 genera, 2 subfamilies
Trombiculinae	4,482 individuals; 96 species, 8 genera
*Leptotrombidium*	Leptotrombidium scutellare** (1,535); *L*. *sinicum* (151); *L*. *parapalpale* (19); *L*. *eothenomydis* (114); *L*. *hiemalis* (1); *L*. *rusticum* (74); *L*. *shuqui* (1); *L*. *wangi* (102); *L*. *densipunctatum* (530); *L*. *yongshengense* (32); *L*. yui* (67); *L*. *xiaguanense* (1); *L*. imphalum* (6); *L*. *gongshanense* (58); *L*. *spicanisetum* (1); *L*. *cuonae* (1); *L*. *deplanoscutum* (1); *L*. *lianghense* (13); *L*. kaohuense** (16); *L*. *qujingense* (5); *L*. *dianchi* (6); *L*. *jinmai* (1); *L*. *longimedium* (12); *L*. akamushi* (2); *L*. *alpinum* (107); *L*. *trapezoidum* (1); *L*. *allosetum* (3); *L*. pallidum* (5); *L*. *sinotupaium* (1); *L*. *hsui* (2); *L*. *robustisetum* (12); *L*. *biluoxueshanense* (20); *L*. *cangjiangense* (15); *L*. *hupeicum* (1); *L*. *apodemi* (4); *L*. *intermedium* (3); *L*. *biji* (5); *L*. *apodevrieri* (3); *L*. *suense* (12); *L*. *kitasatoi* (1); *L*. *huangdi* (1); *L*. *zhongdianense* (30); *L*.*bishanense* (5); *L*. *yunlingense* (219); *L*. *ejingshanense* (5); *L*. *baoshui* (68); *L*. *laojunshanense* (285); *L*. *caudatum* (1); *L*. *xinjiangense* (1); *L*. *jianshanense* (3); *L*. *saltuosum* (15); *L*. *sexsetum* (1); *L*. rupestre* (41); *L*.*bambicola* (4); *L*. *myotis* (1); *L*. *subintermedium* (4); *L*. *liaoji* (16); *L*. *dihumerale* (22); *L*. *rattistae* (1); *L*. *linhuaikongense* (5); *L*. *miyajimai* (1); *L*.*fujianense* (4); *L*. *burnsi* (1); *L*. insulare** (3); *L*. *gemiticulum* (4); *L*. *yunnanense* (11); *L*. *rufocanum* (1); *L*. *huangchuanense* (11); *L*. *bayanense* (6).
*Trombiculindus*	*Trombiculindus alpinus* (1); *T*. *yunnanus* (3); *T*. *cuneatus* (1); *T*. *bambusoides* (13); *T*. *nujiange* (9).
*Neotrombicula*	*Neotrombicula deqinensis* (11); *N*. *japonica* (5); *N*. *tongtianhensis* (65); *N*. *aeretes* (212); *N*. *vulgaris* (5); *N*. *microtomici* (72); *N*. *microti* (255); *N*. *pomeranzori* (1); *N*. *sinica* (1).
*Microtrombicula*	*Microtrombicula nadchatrami* (4).
*Doloisia*	*Doloisia brachypus* (2); *D*. *taishanensis* (2); *D*. *furcipelta* (1).
*Ascoschoengastia*	Ascoschoengastia indica* (1); *A*. *yunnanensis* (69); *A*. *leechi* (13); *A*. *rattinorvegici* (1); *A*. *menghaiensis* (4).
*Herpetacarus*	*Herpetacarus aristoclavus* (1); *H*. *spinosetosus* (7); *H*. *tenuiclavus* (8).
*Euschoengastia*	*Euschoengastia weifangensis* (1).
Gahrliepiinae	1,283 individuals; 27 species, 4 genera
*Walchia*	*Walchia pacifica* (2); *W*. *parapacifica* (4); *W*. *micropelta* (20); *W*.chinensis* (7); *W*. *kor* (6); *W*. *enode* (7); *W*. *sheensis* (4); *W*. *ewingi* (66).
*Schoengastiella*	*Schoengastiella ligula* (195).
*Gahrliepia*	*Gahrliepia zhongwoi* (2); *G*. *longipedalis* (32); *G*. *radiopunctata* (2); *G*. *octosetosa* (1); *G*. *latiscutata* (2); *G*. *lengshui* (8); *G*. *yangchenensis* (27); *G*. *deqinensis* (6); *G*. *tenuiclava* (1); *G*. *tenella* (1); *G*.*chekiangensis* (16); *G*. *yunnanensis* (5); *G*. *chungkingensis* (6); *G*. *megascuta* (21); *G*. *madun* (3); *G*. *linguipelta* (38); *G*. *fimbriata* (792).
*Intermedialia*	*Intermedialia hegu* (9).
Leeuwenhoekiidae	27 individuals; 4 species, 3 genera, 1 subfamily
Leeuwenhoekiinae	27 individuals; 4 species, 3 genera
*Odontacarus*	*Odontacarus tetrasetosus* (12).
*Chatia*	*Chatia maoyi* (2); *C*. *alpine* (1).
*Shunsennia*	*Shunsennia scabrisetosa* (12).
Unidentified species	90 individuals
Total chigger mites	5,882 individuals; 127 species, 15 genera, 3 subfamilies, 2 families

** Main vectors of scrub typhus in China

* Potential vectors of scrub typhus and hemorrhagic fever with renal syndrome (caused by hanta virus; also called “epidemic hemorrhagic fever, EHF” in China)

**Table 5:** The comparative analysis of chigger mites collected from small mammal hosts in MUL and CFL areas (2001–2015).

		MUL			CFL		
Families		2			2		
Genera		15			12		
Species (*S*)		127			86		
Individuals		5,882			17,742		
Shannon–Wiener diversity index (*H*)		3.057			2.506		
Dominant chigger mite species	Name	*L*. *scutellare*	*G*. *fimbriata*	*L*. *densipunctatum*	*A*. *indica*	*L*. *deliense*	*W*. *micropelta*
	Individuals	1,535	792	530	4,580	4,164	2,156
	*C*_*r*_ (%)	26.097	13.465	9.011	25.814	23.470	12.152

In CFL with lower biodiversity in the south of Yunnan, a total of 17,742 chigger mites were collected from 1,112 small mammal hosts, and they were identified as 86 species, 12 genera and 3 subfamilies in two families. The subfamily Trombiculinae had the most abundant species and individuals (59 species in 8 genera with 14,472 individuals), and Gahrliepiinae came next (26 species in 3 genera with 2,886 individuals). Leeuwenhoekiinae had the least species and individuals (one species in one genus with only 16 individuals) (Tables 5 and 6). Three species of chigger mite (*A*. *indica*, *L*. *deliense* and *W*. *micropelta*) were the most dominant species, and their constituent ratios (*C*_*r*_) were 25.81%, 23.47% and 12.15% respectively (Table 5). As showed in Table 5, both the species diversity (*S* = 127) and community diversity (*H* = 3.057) of chigger mites in MUL were much higher than in CFL (*S* = 86; *H* = 2.506).

**Table 6:** Chigger mites collected and identified in CFL from 2001 to 2015.

Taxonomic taxa of chigger mites	Collected individuals and species of chigger mites (The figures in the brackets are the collected individuals for each mite species)
Trombiculidae	17,358 individuals; 85 species, 11 genera, 2 subfamilies
Trombiculinae	14,472 individuals; 59 species, 8 genera
*Leptotrombidium*	Leptotrombidium scutellare** (979); *L*. *sinicum* (238); *L*. *eothenomydis* (28); *L*. *hiemalis* (181); *L*. *rusticum* (91); *L*. *shuqui* (32); *L*. *wangi* (379); *L*. *densipunctatum* (3); *L*. *yongshengense* (70); *L*. yui* (38); *L*. deliense** (4164); *L*. *xiaguanense* (45); *L*. rubellum** (654); *L*. imphalum* (752); *L*. *gongshanense* (45); *L*. *spicanisetum* (35); *L*. *cuonae* (2); *L*.*yuebeinse* (5); *L*. *deplanoscutum* (22); *L*. *lianghense* (143); *L*. *quadrifurcatum* (2); *L*. kaohuense** (4); *L*. *xiaowei* (2); *L*. *chuanxi* (1); *L*. *qujingense* (2); *L*. *nudisensillum* (1); *L*. akamushi* (58); *L*. *alpinum* (7); *L*. *trapezoidum* (7); *L*. *allosetum* (1); *L*. pallidum* (6); *L*. *sinotupaium* (20); *L*. *hsui* (41); *L*. *kunmingense* (9); *L*. *robustisetum* (3); *L*. *guzhangense* (1).
*Trombiculindus*	*Trombiculindus yunnanus* (6); *T*. *cuneatus* (11); *T*. *bambusoides* (2).
*Microtrombicula*	*Microtrombicula munda* (309).
*Eutrombicula*	*Eutrombicula hirsti* (49); *E*. *wichmanni* (16).
*Helenicula*	*Helenicula kohlsi* (1); *H*. *simena* (2); *H*. *hsui* (1).
*Doloisia*	*Doloisia sinensis* (1); *D*. *brachypus* (33); *D*. *moica* (26); *D*. *manipurensis* (6).
*Ascoschoengastia*	Ascoschoengastia indica* (4580); *A*. *yunwui* (124); *A*. *petauristae* (23); *A*. *yunnanensis* (86); *A*. *leechi* (318); *A*. *audyi* (3); *A*. *montana* (1); *A*. *menghaiensis* (773).
*Walchiella*	*Walchiella notiala* (25); *W*. *yingjiangensis* (5).
Gahrliepiinae	2,886 individuals; 26 species, 3 genera
*Walchia*	*Walchia parapacifica* (10); *W*. *micropelta* (2156); *W*. chinensis* (340); *W*. *kor* (23); *W*. *minuscuta* (66); *W*. *turmalis* (80); *W*. *Chuanica* (61); *W*. *erana* (1); *W*. *kritochaeta* (2); *W*. *rustica* (56); *W*. *ewingi* (14).
*Schoengastiella*	*Schoengastiella ligula* (14).
*Gahrliepia*	*Gahrliepia meridionalis* (3); *G*. *zhongwoi* (23); *G*. *longipedalis* (2); *G*. *silvatica* (7); *G*. *banyei* (4); *G*. *radiopunctata* (2); *G*. *octosetosa* (10); *G*. *latiscutata* (1); *G*. *lengshui* (4); *G*. *yangchenensis* (1); *G*. *deqinensis* (1); *G*. *tenuiclava* (3); *G*. *xiaowoi* (1); *G*. *tenella* (1).
Leeuwenhoekiidae	16 individuals; 1 species, 1 genus, 1 subfamily
Leeuwenhoekiinae	16 individuals; 1 species, 1 genus
*Chatia*	*Chatia maoyi* (16).
Unidentified species	368 individuals
Total chigger mites	17,742 individuals; 86 species, 12 genera, 3 subfamilies, 2 families

** Main vectors of scrub typhus in China

* Potential vectors of scrub typhus and hemorrhagic fever with renal syndrome (caused by hanta virus; also called “epidemic hemorrhagic fever, EHF” in China)

### Distribution difference of vector mites in two types of landscapes

**Fig 2:**
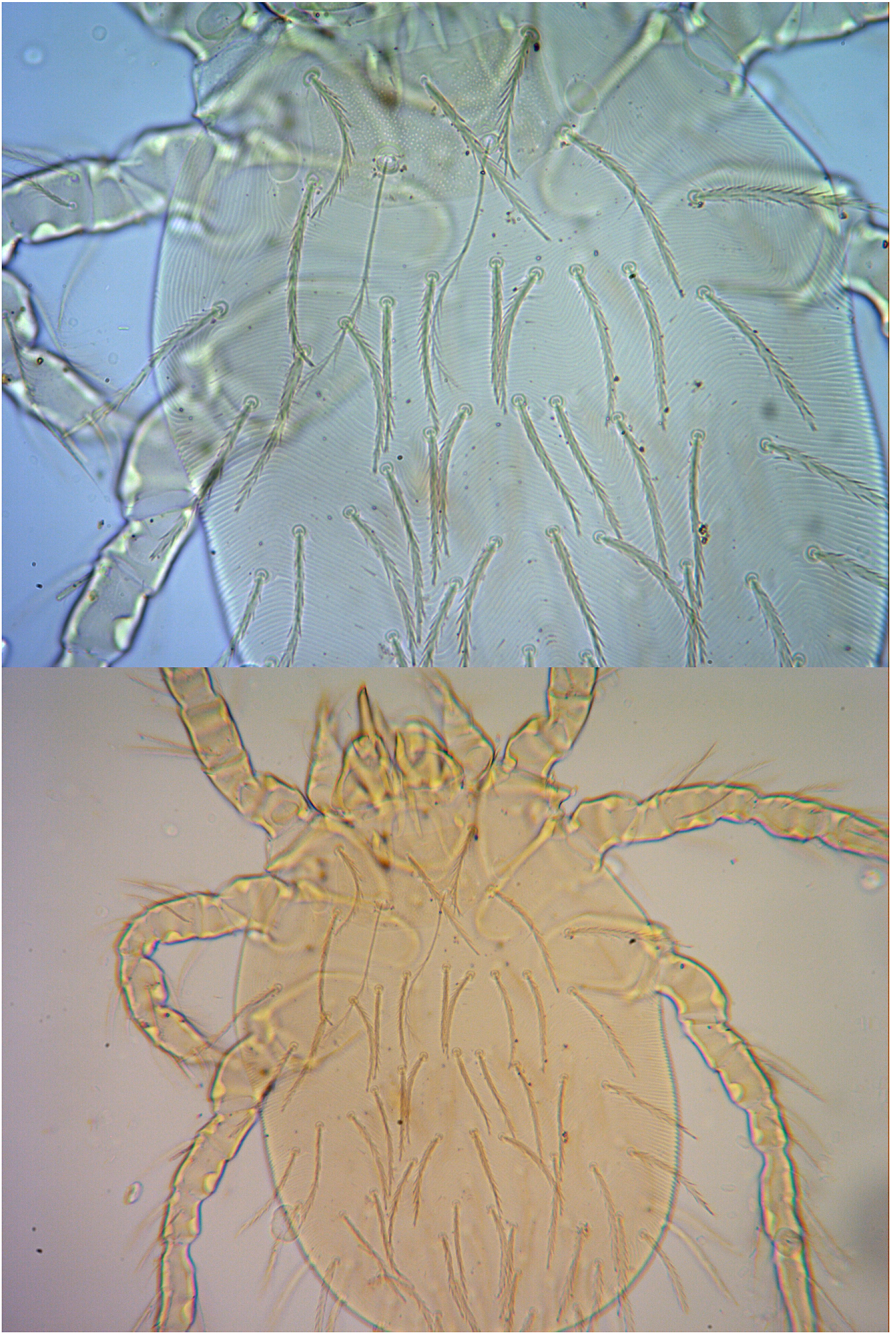
The photos of *Leptotrombidium scutellare*. (a) Complete picture (under microscope: 10×20); (b) Scutum and dorsal setae (under microscope: 10×40).

**Fig 3:**
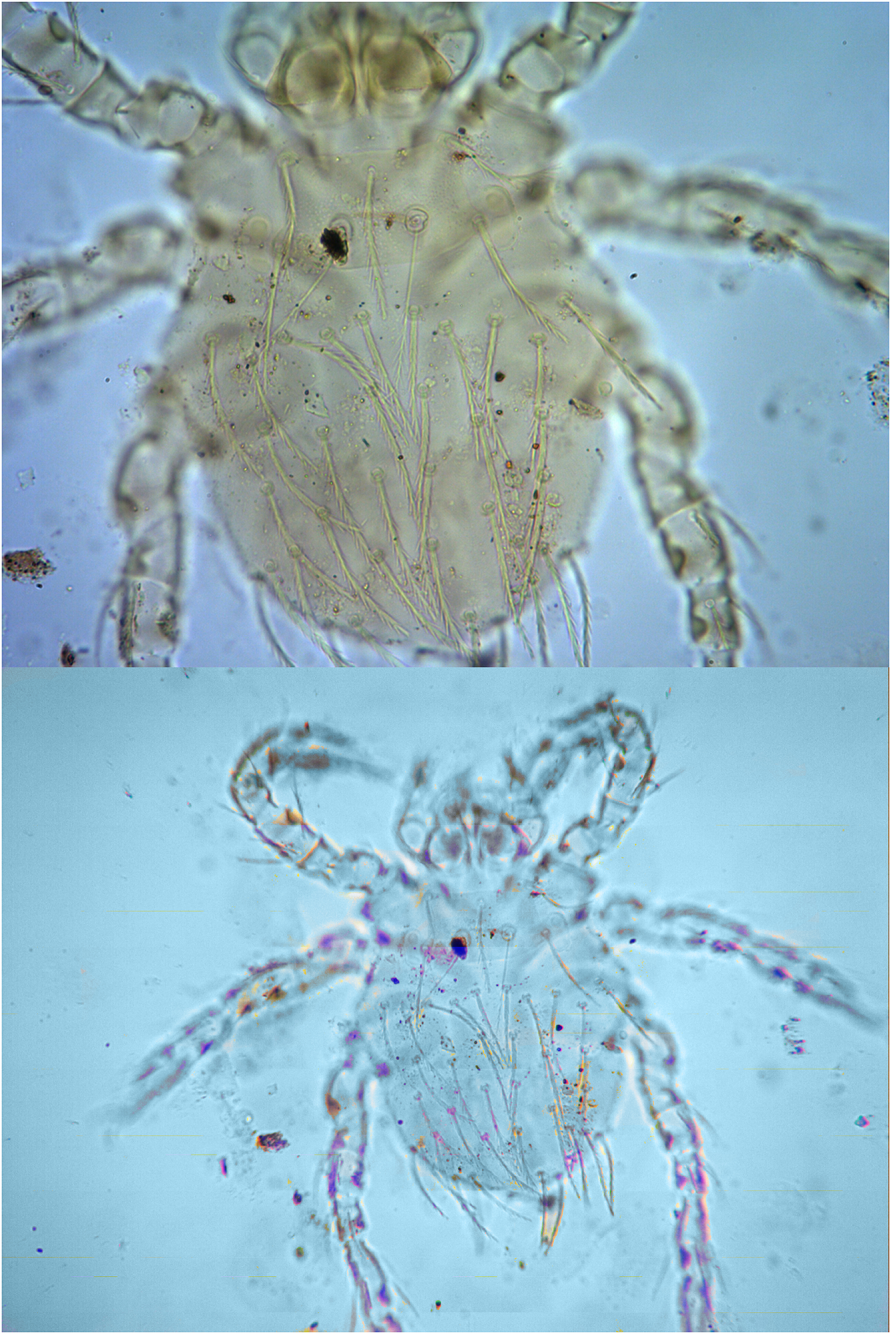
The photos of *Leptotrombidium deliense*. (a) Complete picture (under microscope: 10×20); (b) Scutum and dorsal setae (under microscope: 10×40).

As showed in Tables 4 and 6, the species of chigger mites marked with “**” are some main vectors of scrub typhus in China, and the other mite species marked with “*” are some potential vectors of scrub typhus and hemorrhagic fever with renal syndrome (caused by hanta virus) in China. Of the six main vectors of scrub typhus in China, five vector species were found in the two landscapes of Yunnan province. These five species are *L*. *scutellare*, *L*. *deliense*, *L*. *rubellum*, *L*. *kaohuense* and *L*. *insulare*. Of these five vector species, *L*. *scutellare* and *L*. *deliense* are the dominant mite species (Figs 2 and 3). The only exception was *L*. *jishoum* (one main vector of tsutsugamushi disease in China) which was not collected from the two landscapes in this investigation. A total of 10 vector species or potential vector species of chigger mites were found in each landscape, but the dominant vector species and their distribution were very different. As a main and powerful vector (*L*. *deliense*) or an important potential vector (*A*. *indica*) of tsutsugamushi disease in China, *L*. *deliense* and *A*. *indica* were the dominant chigger mites in CFL (*Cr* = 25.81% for *A*. *indica*; *C*_*r*_ = 23.47% for *L*. *deliense*). Another vector of tsutsugamushi disease and hemorrhagic fever with renal syndrome in some parts of China, *L*. *scutellare* was the dominant chigger mite in MUL (*Cr* = 26.09%). The total percentage of *L*. *deliense* and *A*. *indica* (*Cr* = 25.81% + 23.47% = 49.28%) in CFL was much higher than that of *L*. *scutellare* (*Cr* = 26.09%) in MUL (Table 5).

### Infestation difference of vector mites on small mammal hosts in two types of landscapes

The overall infestations of chigger mites on all the small mammals were different in two types of landscapes. There were much more individuals of chigger mites collected in CFL (17,742) than in MUL (5,882). The overall prevalence (*P* = 38.40%), mean abundance (*MA* = 15.96 mites/per host) and mean intensity (*MI* = 41.55 mites/per host) in CFL were much higher than those in MUL (*P* = 19.96%, *MA* = 1.85 mites/per host, *MI* = 9.28 mites/per host) (Table 7). The main vector species of chigger mites on their main rodent hosts also showed a higher prevalence (*P*), mean abundance (*MA*) and mean intensity (*MI*) in CFL than in MUL. As showed in Table 7 for example, the prevalence (*P* = 14.57%), mean abundance (*MA* = 4.30) and mean intensity (*MI* = 29.49) of *L*. *deliense* on its dominant host species (*R*. *tanezumi*) were high in CFL. In comparison with *L*. *deliense* on *R*. *tanezumi* in CFL, the prevalence (*P*) of *L*. *scutellare* was lower on one of its dominant host species *A*. *chevrieri* (*P* = 3.41%) in MUL. The mean abundance (*MA*) and mean intensity (*MI*) of *L*. *scutellare* on its two dominant host species (*E*. *miletus* and *A*. *chevrieri*) were all lower in MUL (*MA* = 2.47 and *MI* = 9.77 on *E*. *miletus*; *MA* = 0.13 and *MI* = 3.71 on *A*. *chevrieri*). The prevalence (*P*) of *L*. *scutellare* on *E*. *miletus* (one of its dominant host species), however, was higher (*P* = 25.30%) than that of *L*. *deliense* on *R*. *tanezumi* in CFL (*P* = 14.57%) and this is an exception.

**Table 7:** The overall infestations of chigger mites and the infestations of vector mites on their dominant small mammal hosts in two types of landscapes.

	MUL			CFL	
	Overall infestation of chiggers	*L*. *scutellare* on *E*. *miletus*	*L*. *scutellare* on *A*. *chevrieri*	Overall infestation of chiggers	*L*. *deliense* on *R*. *tanezumi*
*P*	19.96%	25.30%	3.41%	38.40%	14.57%
*MA*	1.85	2.47	0.13	15.96	4.30
*MI*	9.28	9.77	3.71	41.55	29.49

*P* = Prevalence; *MA* = Mean abundance; *MI* = Mean infestation

### Beta-diversity measurement of small mammal hosts and chigger mites in two types of landscapes

Cody diversity index was used to analyze the beta-diversity (β diversity) of small mammal hosts and chigger mites in MUL and CFL landscape. From low latitude to high latitude of investigation counties in MUL and CFL landscape, the β diversity of small mammal hosts and chigger mites in MUL landscape were higher than that in CFL landscape (Table 8).

**Table 8:** Comparison on Beta-diversity of small mammal hosts and chigger mites between MUL and CFL.

County (low to high latitude)	MUL		County (low to high latitude)	CFL	
	Hosts	Chigger mites		Hosts	Chigger mites
M1	10.5	20	C1	6.5	14
M2	14.5	27.5	C2	5	23.5
M3	14	31	C3	2	24
M4	10.5	18.5	C4	5	22.5
M5	9.5	26	C5	5	27
$\bar{\text{X}}$	11.8±2.2804	24.6±5.2369	$\bar{\text{X}}$	4.7±1.6432	22.2±4.8811

M1: Fugong→Lijiang; M2: Lijiang→Weixi; M3: Weixi→Gongshan; M4: Gongshan→Xianggelila;

M5: Xianggelila→Deqin. C1: Mengla→Menghai; C2: Menghai→Jinghong; C3: Jinghong→Puer;

C4: Puer→Ninger; C5: Ninger→Yuanjiang.

## Discussion

Small mammals usually involve five orders: Rodentia, Insectivora, Scandentia, Lagomorpha and Chiroptera. Of the five orders, Rodentia (rodents) are the major part with about 2000 species recorded in the world [1]. Different from other categories of small mammals, the majority of rodents (rats, mice and voles, etc.) are pests and they are harmful to agriculture and forestry. Besides destroying crops and forests in agriculture and forestry, some rodents are of medical importance and they can be reservoir hosts and infection sources of some zoonoses [3–6]. Althogh many rodent species can be the potential reservior hosts of some zoonoses, *R*. *tanezumi* is the most important reservior host and infection source of some zoonoses (such as plague, hemorrhagic fever with renal syndrome, scrub typhus and leptospirosis, etc.) in Yunnan province, and *E*. *miletus* and *A*. *chevrieri* come next [3–6]. Six species of chigger mites (*L*. *deliense*, *L*. *scutellare*, *L*. *rubellum*, *L*. *kaohuense*, *L*. *insulare*, *L*. *jishoum*) have been proven to be the main vectors of tsutsugamushi disease in China [13, 41–44], and two of them (*L*. *deliense* and *L*. *scutellare*) are very abundant and wide distributed in Yunnan province [43, 45] (Figs 2 and 3). Besides *L*. *scutellare* has been proved to be the potential vector of hanta virus [12, 46]. About six mite species (*A*. *indica*, *L*. *yui*, *L*. *imphalum*, *L*. *akamushi*, *L*. *pallidum*, *L*. *rupestre*) are suspected to be the potential vectors of tsutsugamushi disease [44, 47, 48].

The distribution of small mammals and their ectoparasitic chigger mites, especially the reservoir hosts and vectors of zoonoses, would influence the spread and incidence of the related zoonoses [49, 50]. As showed in the results of this paper, the species diversity of small mammals was much higher in MUL than in CFL, but more rodents were found in CFL with a higher percentage (Tables 2 and 3). The percentage of the main zoonosis reservoir host (*R*. *tanezumi*) in CFL was much higher than the reservoir hosts (*E*. *miletus* and *A*. *chevrieri*) in MUL (Table 3). Much more species of chigger mites were collected in MUL with a higher species diversity (*S* = 127) than in CFL (*S* = 86) (Tables 4, 5 and 6). Although there were a total of 10 vector species or potential vector species of chigger mites found in each type of landscapes, but the dominant vector species and their percentage were different (Tables 4, 5 and 6). As two dominant species of chigger mites in the cultivated landscape, the total percentage of *L*. *deliense* and *A*. *indica* (*Cr* = 25.81% + 23.47% = 49.28%) in CFL was much higher than that of *L*. *scutellare* (*Cr* = 26.09%) in MUL (Table 5). The infestation of vector mite species on small mammal hosts was more severe in CFL with higher prevalence (*P*), mean abundance (*MA*) and mean intensity (*MI*) than that (infestation) in MUL. The results of the present paper suggest that landscapes with different biodiversity influence the distribution of small mammals and their ectoparasitic chigger mites. A complex landscape with higher biodiversity seems to decrease the intensity of the main reservior hosts and vector species, and a simple landscape with lower biodiversity may benefit the occurrence of the main reservior hosts and vector species [15, 18, 19].

In the present investigation, MUL is situated in the famous “Three Parallel Rivers Region” on the eastern Tibetan Plateau, where three rivers (Jinshajiang, Lancangjiang, and Nujiang rivers) are parallel to each other, flowing from the northwest towards the southwest. The Three Parallel Rivers Region has been attracting a great attention of the world because of its unique geological history on the Asian continent, rich biodiversity and fascinating ethnic cultures [28, 30]. The MUL in the Three Parallel Rivers Region is a typically complex landscape with higher biodiversity. Far from MUL, the selected six counties in the south of Yunnan province have a wide realm of flat-like farmlands with abundant crops, which forms CFL, a typically simple landscape with lower biodiversity. Besides a simple landscape and lower biodiversity, CFL in the south of Yunnan have warm and humid climate, which may be beneficial to the growth and reproduction of chigger mites [7].

Beta diversity is an important component of biological diversity, measuring compositional change in species assemblages across temporal and spatial scales. Beta diversity concerns not only a number of ecological and evolutionary issues [51], but can also guide the selection of protected areas and help to optimize conservation networks [52]. It has thus become a hot topic in biodiversity research in recent years. Researchers have used various measures and analytical methods to investigate patterns of beta diversity and its underlying mechanisms for various taxa and in different regions. Whittaker introduced the term beta diversity in 1960, but defined it vaguely [53]. As the concept of beta diversity evolved, a high variety of measures were developed to quantify the concept. In present paper, we used Cody diversity index to analyze the beta diversity of small mammal hosts and chigger mites [40]. The results of the beta diversity further supported that there was lower species diversity of small mammals and their ectoparasitic chigger mites, but higher individual abundance of main rodent reservoir species and main vector mite species in the simple landscape (CFL) in comparison with in the complex landscape (MUL) (Table 8).

According to the results in the present paper, the species diversity of small mammals and their ectoparasitic chigger mites in the simple landscape (CFL) was much lower, but the percentage of reservoir rodent hosts and vector mites of zoonoses were prominently higher and the infestation of vector mites on their hosts were much more severe in comparison with those in the complex landscape (MUL). The simple landscape with lower biodiversity, together with warm and humid climate, seems to have caused the quantity increase of some reservoir rodent hosts and vector chigger mites with the decrease of their species diversity. By contrast the complex landscape with higher biodiversity seems to have depressed some reservoir rodent hosts and vector chigger mites. The decrease of species diversity and the quantity increase of reservoir rodent hosts and vector chigger mites would likely increase the potential risk of the spread of tsutsugamushi diseases and some other related zoonoses.

Some studies have previously revealed that there are some links between ecological environments (including landscapes and biodiversity, etc.) and human health. The fragmentation of forests, wetlands destruction and some other habitat destruction or modification would lead to the loss of biodiversity. The destruction of ecological environment and biodiversity loss would increase the human contact rates with various pathogens and disease vectors, which can finally increase the spread and incidence of some diseases [14–24]. The results in our study revealed that there were much more individuals and higher percentage of the main zoonosis reservoir rodent hosts and the main vector chigger mites in the simple landscape with lower biodiversity (CFL) and this would likely increase the potential risk of the spread of the related zoonoses. Our results support the previous opinions related to the relationship between ecological environments and human health. The complex landscape with higher biodiversity may depress the reservoir rodent hosts and vector mites of zoonoses, and therefore it is of medical significance to preserve the biodiversity in the environments and prevent them from being destroyed.

## Appendix

(The full list of species that were considered "pests" and the number that were euthanized in parentheses, MUL and CFL included)

*Apodemus draco* (641)
*Apodemus chevrieri* (411)
*Apodemus deninsulae* (104)
*Apodemus sylvaticus* (15)
*Apodemus latronum* (233)
*Apodemus agrarius* (4)
*Rattus tanezumi* (981)
*Rattus nitidus* (104)
*Rattus norvegicus* (88)
*Rattus sladeni* (96)
*Rattus bowersi* (16)
*Niviventer confucianus* (245)
*Niviventer fulvescens* (149)
*Niviventer andersoni* (10)
*Niviventer eha* (1)
*Niviventer excelsior* (2)
*Mus caroli* (1)
*Mus pahari* (11)
*Mus musculus* (8)
*Micromys minutus* (3)
*Vernaya fulva* (1)
*Vandeleuria oleracea* (1)
*Bandicota indica* (1)
*Eothenomys miletus* (506)
*Eothenomys eleusis* (2)
*Eothenomys proditor* (60)
*Eothenomys custos* (56)
*Suncus murinus* (38)
*Crocidura attenuata* (24)
